# The accuracy of ultrasound viscoelastic imaging in evaluating renal fibrosis and interstitial inflammation of chronic kidney disease

**DOI:** 10.1186/s12882-026-04762-y

**Published:** 2026-01-30

**Authors:** Lan Zeng, Min Lu, Jeong Min Lee, Haoyue Niu, Ying Fu, Yao Yao, Wen Tang, Jie Jiang, Jiuyi Ma, Ligang Cui

**Affiliations:** 1 Department of Ultrasound, Peking University Third Hospital, Beijing, China; 2 Department of Pathology, School of Basic Medical Sciences, Peking University Third Hospital, Peking University Health Science Center, Beijing, China; 3 Department of Radiology, Seoul National University Hospital, Seoul, Korea; 4 Department of Pathology, Peking University Third Hospital, Beijing, China; 5 Department of Nephrology, Peking University Third Hospital, Beijing, China

**Keywords:** Chronic kidney disease, Shear wave elastography, Fibrosis, Inflammation

## Abstract

**Background:**

Accurate evaluation of renal fibrosis and interstitial inflammation grades is necessary to improve the prognosis of patients with chronic kidney disease (CKD). However, non-invasive and accurate diagnostic methods are lacking. In this regard, shear wave elastography, which measures tissue viscosity and elasticity, may be a promising alternative. Therefore, this study aimed to evaluate the diagnostic performance of individual viscoelastic parameters, combined viscoelastic parameter models, and combined clinical parameter models in assessing the grades of renal fibrosis and interstitial inflammation.

**Methods:**

This prospective study recruited 22 and 47 patients with CKD in the reproducibility and validity trials, respectively, between October 2023 and June 2024. The mean elasticity (Emean), maximum elasticity (Emax), minimum elasticity (Emin), and mean viscosity (Vmean) were obtained using two-dimensional shear-wave elastography (2D-SWE). The biopsy specimens were assessed to determine the grades of renal fibrosis and interstitial inflammation. Additionally, clinical data, including protein-to-creatinine ratio, and estimated glomerular filtration rate (eGFR), were obtained from the patients’ medical records. Multiple logistic regression models were constructed, including viscoelastic models and clinical models, and their diagnostic performance was evaluated using receiver operating characteristic curves.

**Results:**

The Emean, Emax, Emin and Vmean demonstrated good to excellent intra-observer consistency (ICCs = 0.839–0.929, *p* < 0.001). The area under the curve (AUC) of Emax combined with Vmean was not significantly superior to that of Emax alone in distinguishing fibrosis grades (0.740 [95% confidence interval (CI): 0.591–0.857] vs. 0.737 [95% CI: 0.589–0.855], *p* > 0.05). In contrast, Emax combined with eGFR demonstrated an AUC of 0.902 (95% CI: 0.780–0.969), a sensitivity of 93.3%, and a specificity of 84.4% in distinguishing fibrosis grades. Individual Vmean achieved an AUC of 0.919 (95% CI: 0.801–0.978), a sensitivity of 100%, and a specificity of 86.1% in differentiating between the grades of renal interstitial inflammation.

**Conclusions:**

A combination of Emax and eGFR was useful for detecting severe renal fibrosis. Additionally, Vmean was identified as a potentially useful parameter for differentiating interstitial inflammation grades, although this finding requires validation in larger cohorts given the limited samples with severe interstitial inflammation.

**Trial registration (retrospectively registered):**

NCT06961162. Registration date: 2025-04-29.

## Introduction

Chronic kidney disease (CKD) is a major chronic non-communicable disease, with a global prevalence of approximately 9–10%, resulting in over 1.4 million deaths per year [1, 2]. Moreover, the number of deaths due to CKD is expected to increase to 2.2–4 million by 2040 [3]. Consequently, accurate assessment and stratification of patients with CKD are crucial to impede disease progression and improve patient prognosis [4].

Renal fibrosis is a common pathological manifestation of CKD, which is independent of its underlying etiology and is considered the most important independent risk factor for CKD prognosis [2]. Interstitial inflammation is a key characteristic that leads to progressive renal fibrosis [5]. Inflammation is associated with endothelial dysfunction and activation of glomerular and tubular epithelial cells, with the consequent release of inflammatory molecules further recruiting immune cells into damaged kidneys [2, 5]. Currently, renal biopsy is the gold standard for diagnosing renal fibrosis and interstitial inflammation [6]. However, this is an invasive procedure that may result in infection, bleeding, and arteriovenous fistulas; therefore, it is impractical for frequent long-term monitoring [7].

Two-dimensional shear wave elastography (2D-SWE) is an advanced ultrasound technique that maps tissue stiffness in real-time. Although previous studies have shown that elasticity plays an important role in the evaluation of liver fibrosis [8, 9], the value of elasticity in the assessment of renal fibrosis remains controversial owing to the deep location of the kidney, the anisotropy of the kidney, renal hemodynamics, and other unknown reasons [10]. Moreover, various studies have reported positive, negative, and no correlations between renal stiffness and renal fibrosis [11–13]. Furthermore, Richard et al. suggested that as of June 2018, the accuracy of elastography of the kidney parenchyma was inadequate [14]. Consequently, new algorithms and parameters are required to improve the accuracy of renal fibrosis diagnosis.

Recently, advances in 2D-SWE have enabled the assessment of tissue elasticity and viscosity through newly developed quantitative parameters [15]. 2D-SWE generates shear waves within tissues, and records the local shear-wave velocity to estimate tissue stiffness [16]. However, renal tissue exhibits viscoelastic properties, and shear wave propagation in renal tissue is governed by elastic and viscous components [17]. Therefore, in such tissues, shear wave speed varies with frequency, a phenomenon known as shear wave dispersion, which reflects the viscous component of tissue mechanics [18, 19]. Consequently, higher tissue viscosity induces a more pronounced frequency-dependent increase in shear-wave speed, resulting in a higher dispersion slope (DS). Accordingly, the DS, together with viscosity parameters obtained by fitting the shear wave dispersion curve to viscoelastic models, serves as a quantitative indicator of tissue viscosity.

Although recent studies have suggested that the combination of the DS and elasticity does not improve the diagnostic efficiency of liver fibrosis [20], the DS has high value in the assessment of liver necroinflammation [21]. In addition, Yuan et al. indicated that the mean viscosity (Vmean) was positively correlated with the grades of fibrosis and inflammatory cell infiltration in the kidneys [22]. Their combined model of viscoelastic parameters achieved an area under the curve (AUC) of 0.910 for predicting CKD [22]. However, whether viscosity improve diagnostic accuracy for renal fibrosis and interstitial inflammation grades, and whether viscoelastic parameters combined with other clinical parameters enhance diagnostic efficacy of renal fibrosis and interstitial inflammation grades, remain unclear.

Therefore, this study aimed to evaluate the diagnostic performance of individual viscoelastic parameters, combined viscoelastic parameter models, and combined clinical parameter models in assessing the grades of renal fibrosis and interstitial inflammation.

## Methods

### Study population

This study was conducted in accordance with the principles of the Declaration of Helsinki. This prospective study was approved by the Ethics Committee of Peking University Third Hospital (2023–681-01).

In total, 22 patients diagnosed with CKD between October 2023 and February 2024 were enrolled in the reproducibility trial. The inclusion criteria for reproducibility trial were as follows: patients who (1) met the diagnostic criteria for CKD (disease course > 3 months) [4]; and (2) provided informed written consent. The exclusion criteria were as follows: (1) the presence of multiple renal cysts, calculi, hydronephrosis, or masses that could potentially interfere with the analysis conducted using 2D-SWE; (2) distance between the renal cortex and skin surface ≥ 8 cm; and (3) unsuccessful 2D-SWE measurements.

**Fig. 1:**
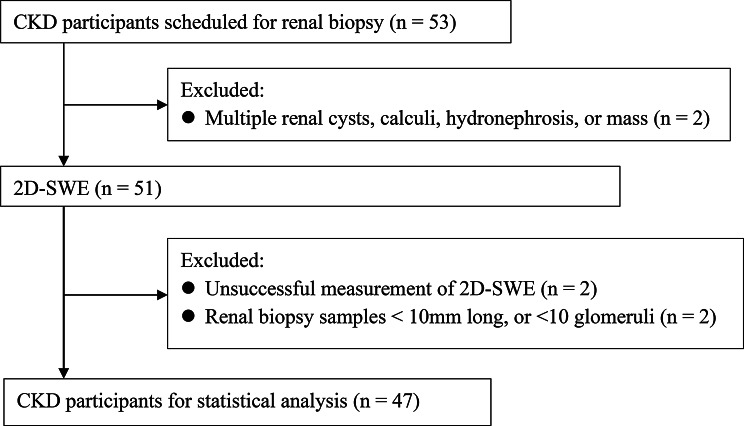
Flowchart illustrating the participant selection process in the validity trial. CKD, chronic kidney disease; 2D-SWE, two-dimensional shear-wave elastography

Additionally, 47 patients diagnosed with CKD between October 2023 and June 2024 at our hospital were enrolled in the validity trial. The inclusion criteria were as follows: patients who (1) met the diagnostic criteria for CKD (disease course > 3 months) [4]; (2) underwent renal biopsy, (3) underwent a 2D-SWE evaluation preceding renal biopsy, and (4) provided informed written consent. The exclusion criteria were as follows: (1) the presence of multiple renal cysts, calculi, hydronephrosis, or masses that could potentially interfere with the analysis conducted through 2D-SWE; (2) distance between the renal cortex and skin surface ≥ 8 cm; (3) unsuccessful 2D-SWE measurements; (4) biopsy specimens deemed inadequate due to insufficient length (< 10 mm) or fewer than 10 glomeruli; and (5) incomplete clinical data. The workflow for patient selection is shown in Fig. 1.

Clinical data, including blood pressure, blood glucose, body mass index (BMI), protein-to-creatinine ratio (PCR), and estimated glomerular filtration rate (eGFR), were obtained from the patients’ medical records.

### Conventional ultrasound and 2D-SWE examination

One day before renal biopsy, a radiologist with five years of experience in abdominal ultrasound, who was blinded to the clinical and pathological results, performed conventional and elastography-based measurements for all patients using a renal software. This included elasticity and viscosity measurements from the Hologic Aixplorer Mach 30 ultrasound system (Aixplorer, Supersonic Imaging, Aix-en-Provence, France) equipped with a C6-1X convex array transducer. The elasticity within the region of interest (ROI) for the tissue was computed by the system using the formula E = 3ρc_s_^2^, where E denotes tissue elasticity in kPa, ρ represents tissue density in kg/m³, and c_s_ indicates shear-wave velocity in m/s. This system also incorporates Vi PLUS technology, which allows users to receive information on tissue shear wave dispersion, which may be used to deduce viscosity [17].

**Fig. 2:**
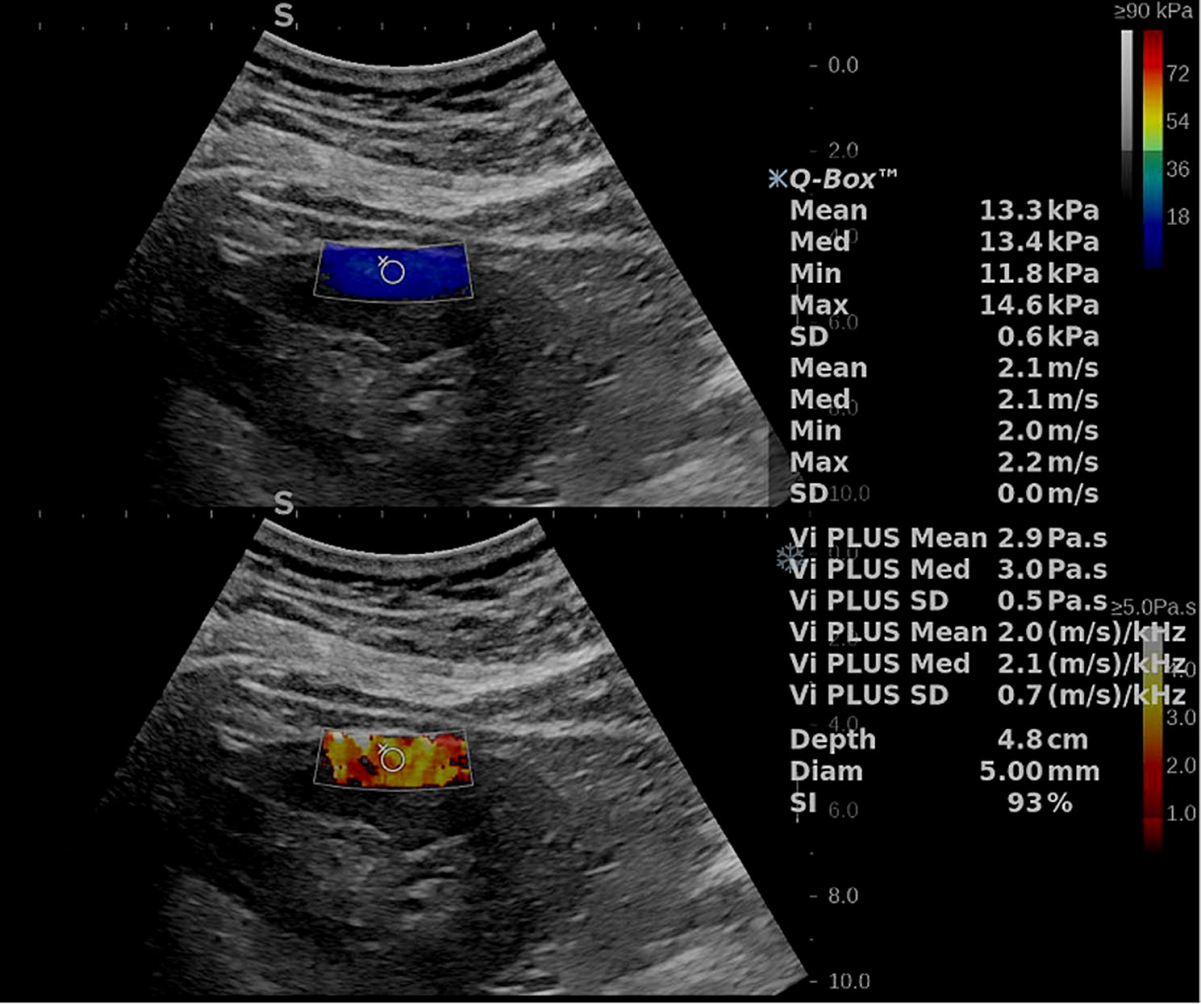
Viscoelasticity map of a patient with CKD. The top image exhibits a color-coded elastogram, while the bottom image shows a color-coded viscosity diagram. CKD, chronic kidney disease

All patients were required to void their bladders before the examination. First, the patients underwent a conventional ultrasound of the right kidney, and the renal longitudinal diameter, renal parenchymal thickness, peak velocity, end-diastolic velocity, and resistive index (RI) of the interlobar artery of the kidney were recorded. Subsequently, under the guidance of conventional ultrasound, viscoelastic measurements were performed in the middle portion of the right kidney with the patients in the prone position. During the measurement, each patient was instructed to exhale normally and then hold their breath at the end of expiration. The image was frozen once it had stabilized, typically within 4–5 s. On the frozen image, a circular ROI (5 mm diameter) was positioned in the outer layer of the renal cortex, adjacent to the renal capsule. The mean elasticity (Emean), maximum elasticity (Emax), minimum elasticity (Emin), Vmean, and DS of the shear wave were recorded (Fig. 2). Six valid measurements were obtained for each kidney, and the median values were calculated. Unsuccessful 2D-SWE measurements were defined as fewer than 6 valid measurements obtained from 10 attempts. A stability index > 85% was deemed as a valid measurement.

### Renal biopsy

All patients underwent a renal biopsy of the right inferior pole of the kidney parenchyma with an 18G biopsy needle from an automatic biopsy gun (Bard Magnum, Covington, GA, USA) under ultrasonic guidance in the prone position. All biopsy samples were fixed in 10% formalin and stained with hematoxylin and eosin, periodic acid − Schiff, Masson’s trichrome, and methenamine silver for further analysis. Two pathologists with 10–30 years of experience, who were blinded to the ultrasonic and clinical results, independently examined the samples under light microscopy. In case of a discrepancy, a consensus was reached through discussion.

In the semi-quantitative pathological scoring system, fibrosis and inflammation are typically graded based on the affected area as none (0%), mild (1–25%), moderate (26–50%), and severe (> 50%). The primary objective of our study was to investigate the diagnostic value of ultrasound viscoelasticity in fibrosis grading. Therefore, a single classification threshold was adopted. Previous studies have shown that the 50% threshold is a critical cutoff for distinguishing different clinical outcomes. Patients with none or mild fibrosis exhibited a similar risk of rapid renal function decline, while those with moderate and severe fibrosis had a 3.0-fold and 21.8-fold increased risk, respectively [23]. Therefore, this study adopted 50% as the unified classification threshold. Patients were divided into two groups based on interstitial fibrosis and tubular atrophy area: mild-to-moderate fibrosis group (0–50%) and severe fibrosis group (> 50%); and based on interstitial inflammation area: mild-to-moderate inflammation group (0–50%) and severe inflammation group (> 50%).

### Statistical analysis

All statistical analyses were performed using the Statistical Package for the Social Sciences software 26.0 software (IBM Inc., Chicago, IL, USA) and MedCalc 19.4 (MedCalc Software Corp, Brunswick, ME, USA). Normally distributed continuous variables are expressed as mean ± deviation and were analyzed using Student’s t test. Non-normally distributed continuous variables are expressed as median (interquartile range) and were analyzed using the Mann–Whitney U test. Categorical variables were analyzed using the chi-squared test. Intra-class correlation coefficients (ICCs) were calculated to evaluate the reliability of ultrasound viscoelastic measurements. ICC was based on absolute agreement, a two-way random model. Univariate and multivariate binary logistic regression analyses were used to filter the variables and construct a model to predict severe fibrosis. The performance of the parameters and models in differentiating between mild-to-moderate and severe fibrosis and mild-to-moderate and severe interstitial inflammation was analyzed using the receiver operating characteristic curve (ROC). The optimal cut-off value was determined based on the Youden index and the corresponding sensitivity and specificity were computed. Delong’s test was used to compare the AUC of the different diagnostic approaches. The sensitivities and specificities of the parameters and models were compared using McNemar’s test. Statistical significance was defined as a two-sides *p* < 0.05.

## Results

### Reliability

The reproducibility trial included 22 patients with CKD, of which 50.0% were men and 50.0% were women, with a mean age of 50.73 ± 16.15 years. The Emean, Emax, Emin and Vmean demonstrated good to excellent intra-observer consistency (ICCs = 0.839–0.929, all *p* < 0.001), while DS showed moderate intra-observer consistency (ICC = 709, *p* = 0.003) (Table 1).

**Table 1:** Intra-observer agreement in renal ultrasound viscoelastic measurement

Parameters	ICC	*p* value
Emean	0.929	< 0.001*
Emax	0.894	< 0.001*
Emin	0.904	< 0.001*
Vmean	0.839	< 0.001*
DS	0.709	0.003*

ICC, intraclass correlation coefficient; Emean, mean elasticity; Emax, maximum elasticity; Emin, minimum elasticity; Vmean, mean elasticity; DS, dispersion slope. * Represents *p* < 0.05

### Patients’ characteristics in the validation trial

A total of 47 patients with CKD were included in the validation trial. Among them, 32 (68.1%) were diagnosed with mild-to-moderate fibrosis, 15 (31.9%) with severe fibrosis, 43 (91.5%) with mild-to-moderate interstitial inflammation, and 4 (8.5%) with severe interstitial inflammation. Post-procedure hemorrhage occurred in 13 (27.7%) patients after renal biopsy.

The severe fibrosis group showed significantly higher values than the mild-to-moderate fibrosis group across the three viscoelastic parameters: Emean (25.67 ± 5.53 kPa vs. 19.37 ± 9.02 kPa, *p* = 0.005), Emax (30.48 ± 4.81 kPa vs. 22.93 ± 9.75 kPa, *p* = 0.001), and Vmean (3.54 ± 0.40 Pa·s vs. 3.16 ± 0.70 Pa·s, *p* = 0.020). Compared to the mild-to-moderate fibrosis group, the severe fibrosis group had significantly lower levels of hemoglobin (116.80 ± 22.96 g/L vs. 133.25 ± 25.27 g/L, *p* = 0.038), and eGFR (46.13 ± 24.64 mL/min/1.73 m² vs. 84.13 ± 32.86 mL/min/1.73 m², *p* < 0.001), and renal interlobar artery end-diastolic velocity (5.33 ± 2.24 cm/s vs. 7.21 ± 2.82 cm/s, *p* = 0.033). The severe interstitial inflammation group had a higher Vmean than the mild-to-moderate interstitial inflammation group (4.06 ± 0.22 Pa·s vs. 3.21 ± 0.62 Pa·s, *p* = 0.009). In contrast, the severe interstitial inflammation group had a lower eGFR than the mild-to-moderate interstitial inflammation group (23.25 ± 13.96 mL/min1.73 m^2^ vs. 76.53 ± 33.00 mL/min1.73 m^2^, *p* = 0.001). The demographic, laboratory, conventional ultrasound, and viscoelastic ultrasound characteristics of patients with CKD are shown in Tables 2 and 3.

**Table 2:** Clinical and Ultrasound features of the mild-to-moderate and severe fibrosis groups

Characteristics	Mild-to-moderate fibrosis	Severe fibrosis	*p* value
	*n* = 32	*n* = 15	
Sex			0.261
Male	18	11	
Female	14	4	
Age (years)	53.50 (47.75,62.00)	58.00 (35.00,64.00)	0.864
BMI (kg/m^2^)	25.86 ± 4.97	25.53 ± 3.65	0.825
Hypertension			0.353
Yes	19	11	
No	13	4	
Diabetes mellitus			0.696
Yes	13	7	
No	19	8	
Laboratory indicators			
Hemoglobin (g/L)	133.25 ± 25.27	116.80 ± 22.96	0.038*
eGFR (mL/min/1.73m^2^)	84.13 ± 32.86	46.13 ± 24.64	< 0.001*
PCR (mg/g)	2443.00 (920.00,3550.50)	4038.00 (1784.00,8013.00)	0.124
Conventional ultrasound parameters			
Renal longitudinal diameter (cm)	10.40 ± 0.99	10.43 ± 1.37	0.936
Renal parenchyma thickness (cm)	0.78 ± 0.13	0.87 ± 0.18	0.058
Peak velocity (cm/s)	20.96 (16.64,25.94)	17.96 (12.70,21.07)	0.068
End-diastolic velocity (cm/s)	7.21 ± 2.86	5.33 ± 2.24	0.028*
RI	0.67 ± 0.07	0.70 ± 0.12	0.402
Viscoelastic parameters			
Emean (kPa)	19.37 ± 9.02	25.67 ± 5.53	0.005*
Emax (kPa)	22.93 ± 9.75	30.48 ± 4.81	< 0.001*
Emin (kPa)	9.40 (4.73,21.18)	16.05 (9.95,19.95)	0.205
Vmean (Pa·s)	3.16 ± 0.70	3.54 ± 0.40	0.020*
DS (m/s/kHz)	2.58 ± 0.50	2.48 ± 0.32	0.441
Etiology			-
IgA nephropathy	7	4	
Membranous nephrology	11	1	
MCN	4	0	
Diabetic nephropathy	4	6	
FPGN	2	0	
FSGS	2	0	
Others	2	4	
CKD stage			-
G1	18	1	
G2	4	3	
G3	9	5	
G4	1	3	
G5	0	1	

BMI, body mass index; eGFR, estimated glomerular filtration rate; PCR, protein-to-creatinine ratio; RI, resistive index; Emean, mean elasticity; Emax, maximum elasticity; Emin, minimum elasticity; Vmean, mean viscosity; DS, dispersion slope. MCN, minimal change nephropathy; FPGN, focal proliferative glomerulonephritis; FSGS, focal segmental glomerular sclerosis; CKD, chronic kidney disease. * Represent *p* value < 0.05

**Table 3:** Clinical and Ultrasound features of the mild-to-moderate interstitial inflammation and severe interstitial inflammation groups

Characteristics	Mild-to-moderate interstitial inflammation	Severe interstitial inflammation	*p* value
	*n* = 43	*n* = 4	
Sex			0.298
Male	28	1	
Female	15	3	
Age (years)	54.00 (46.75,62.25)	49.50 (26.00,70.00)	1.000
BMI (kg/m^2^)	24.82 (22.88,29.27)	29.70 (21.02,30.62)	0.364
Hypertension			1.000
Yes	27	3	
No	16	1	
Diabetes mellitus			0.831
Yes	19	1	
No	24	3	
Laboratory indicators			
Hemoglobin (g/L)	129.98 ± 24.71	106.75 ± 27.75	0.081
eGFR (mL/min/1.73m^2^)	76.53 ± 33.00	23.25 ± 13.96	0.001*
PCR (mg/g)	2694.00 (1004.00,5150.00.00)	4082.50 (1243.50,4574.75)	0.732
Conventional ultrasound parameters			
Renal longitudinal diameter (cm)	10.49 ± 1.03	9.54 ± 1.76	0.099
Renal parenchyma thickness (cm)	0.81 ± 0.15	0.84 ± 0.21	0.657
Peak velocity (cm/s)	20.10 (16.40,24.93)	17.03 (13.42,19.62)	0.196
End-diastolic velocity (cm/s)	6.85 ± 2.70	4.08 ± 2.50	0.055
RI	0.68 ± 0.78	0.74 ± 0.19	0.581
Viscoelastic parameters			
Emean (kPa)	20.82 ± 8.49	27.31 ± 7.63	0.148
Emax (kPa)	24.74 ± 9.22	31.74 ± 5.90	0.188
Emin (kPa)	13.46 ± 8.42	19.24 ± 10.63	0.205
Vmean (Pa·s)	3.21 ± 0.62	4.06 ± 0.22	0.009*
DS (m/s/kHz)	2.56 ± 0.46	2.44 ± 0.18	0.611
Etiology			-
IgA nephropathy	10	1	
Membranous nephrology	12	0	
MCN	4	0	
Diabetic nephropathy	9	1	
FPGN	2	0	
FSGS	2	0	
Others	4	2	
CKD stage			-
G1	19	0	
G2	7	0	
G3	15	1	
G4	2	2	
G5	0	1	

BMI, body mass index; eGFR, estimated glomerular filtration rate; PCR, protein-to-creatinine ratio; RI, resistive index; Emean, mean elasticity; Emax, maximum elasticity; Emin, minimum elasticity; Vmean, mean viscosity; DS, dispersion slope; MCN, minimal change nephropathy; FPGN, focal proliferative glomerulonephritis; FSGS, focal segmental glomerular sclerosis; CKD, chronic kidney disease. * Represent *p* value < 0.05

### Model construction

The multivariate analysis only included Emax due to the high correlation between Emean and Emax and because previous studies have shown that Emax was more effective than Emean in distinguishing renal fibrosis grades [24].

According to the univariate and multivariate analyses, eGFR and Emax successfully predicted severe fibrosis, and eGFR and Vmean successfully predicted severe interstitial inflammation (Tables 2, 3, and 4). Therefore, Model 2, for predicting severe fibrosis, included eGFR and Emax, whereas Model 4, for predicting severe interstitial inflammation, included eGFR and Vmean. Additionally, we constructed Models 1 and 3, by including Emax and Vmean for the diagnosis of fibrosis and interstitial inflammation, respectively, via the logistic regression algorithm, to verify whether the accuracy of the diagnosis of renal fibrosis grades and interstitial inflammation grades was improved by adding viscosity.

**Table 4:** Univariate and multivariate regression analysis of clinical and ultrasonic parameters in predicting severe fibrosis of kidney

Parameters	Univariate analysis		Multivariate analysis	
	OR (95% CI)	*p* value	OR (95% CI)	*p* value
Hemoglobin (g/L)	0.973 (0.947–0.999)	0.046*	1.007 (0.967–1.049)	0.724
eGFR (mL/min/1.73m^2^)	0.960 (0.935–0.985)	0.002*	0.967 (0.936–1.000)	0.047*
End-diastolic velocity (cm/s)	0.744 (0.558–0.992)	0.044*	0.702 (0.413–1.193)	0.702
Emean (kPa)	1.114 (1.014–1.222)	0.024*		
Emax (kPa)	1.129 (1.021–1.248)	0.018*	1.208 (1.032–1.193)	0.018*
Vmean (Pa·s)	3.049 (0.951–9.7776)	0.061		

OR, odds ratio; CI: confidence interval; eGFR, estimated glomerular filtration rate; Emean, mean elasticity; Emax, maximum elasticity; Emin, minimum elasticity; Vmean, mean elasticity. * Represents *p* < 0.05

### Performance of individual parameterss and combined models

**Fig. 3:**
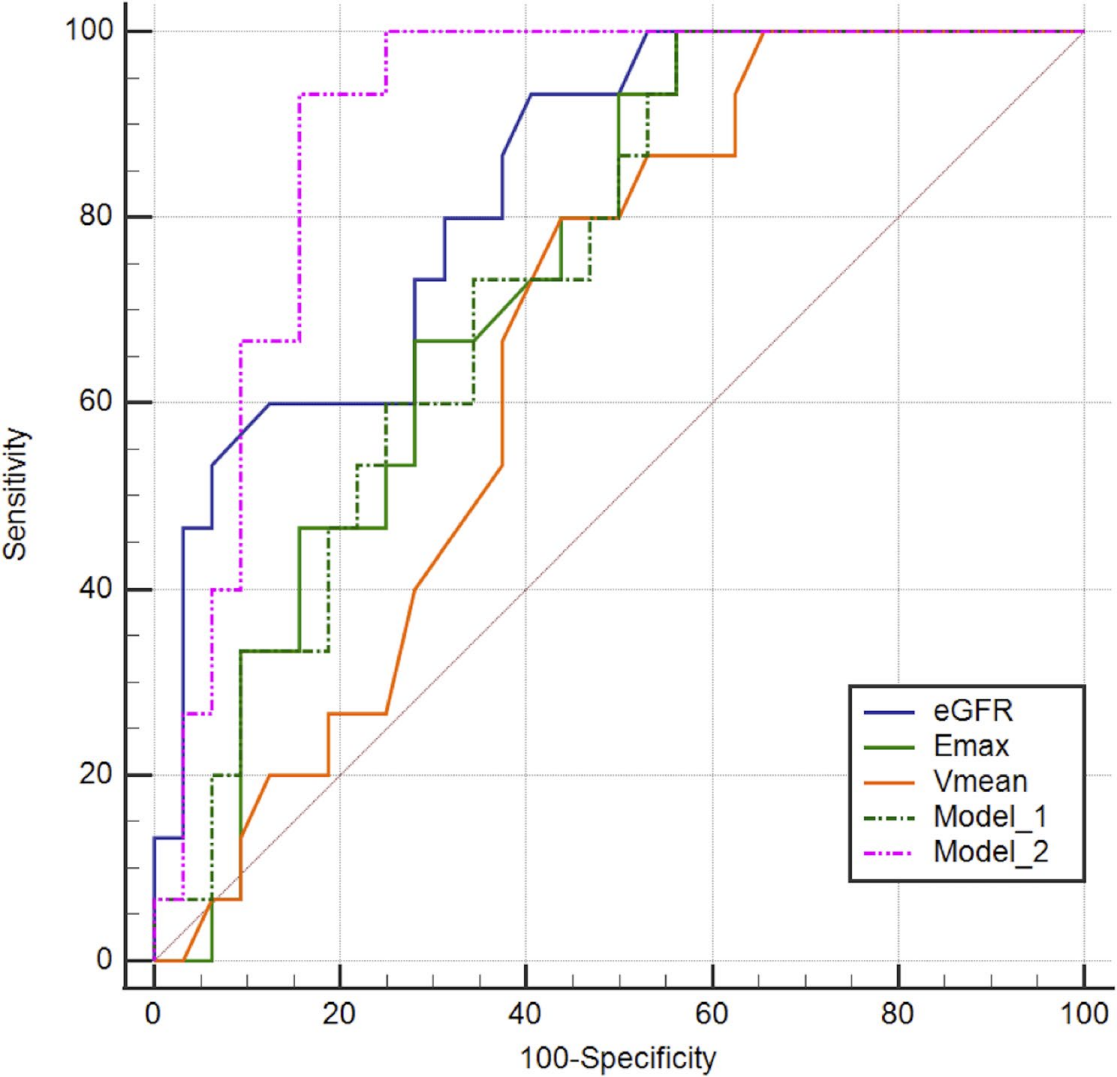
ROC

Compared to the AUC of Emax alone and Vmean alone, Model 1, which combined Emax and Vmean, did not exhibit a significantly improved AUC in predicting severe fibrosis (0.740 [95% confidence interval (CI): 0.591–0.857] vs. 0.737 [95% CI: 0.589–0.855] and 0.740 [95%CI: 0.591–0.857] vs. 0.669 [95%CI: 0.516–0.799], *p* = 0.907 and *p* = 0.319, respectively). The AUC of Model 2 was higher than that of Emax alone [0.902 (95% CI: 0.780–0.969) vs. 0.737 (95% CI: 0.589–0.855), *p* = 0.012]. Although no significant difference was observed in the AUCs between Model 2 and eGFR alone (*p* = 0.161), the specificity of Model 2 was higher than that of eGFR (84.4% vs. 59.4%, *p* = 0.008) (Table 5; Fig. 3).

**Table 5:** Comparison of diagnostic performance in fibrosis between different approaches

Parameters	AUC			Sensitivity			Specificity			Cut-off value
	% (95% CI)	*p* value	*p* value^#^	(%)	*p* value	*p* value^#^	(%)	*p* value	*p* value^#^	
eGFR	0.835 (0.698–0.927)	0.329	0.161	93.3	1.000	1.000	59.4	0.332	0.008*	82.00
Emax	0.737 (0.589–0.855)	0.907	0.012*	100	1.000	1.000	43.8	1.000	< 0.001*	21.90
Vmean	0.669 (0.516–0.799)	0.319	0.003*	80	0.250	0.332	56.3	0.125	< 0.001*	2.75
Model 1	0.740 (0.591–0.857)	-	0.012*	100	-	1.000	43.8	-	< 0.001*	0.15
Model 2	0.902 (0.780–0.969)	-	-	93.3	-	-	84.4	-	-	0.31

*p* value refers to comparison with Model 1; *p* value^#^ refers to comparison with Model 2. Model 1 combined Emax with Vmean, and Model 2 combined Emax with eGFR. AUC, area under the curve; CI, confidence interval; eGFR, estimated glomerular filtration rate; Emax, maximum elasticity; Vmean, mean elasticity. * Represent *p* value < 0.05

**Fig. 4:**
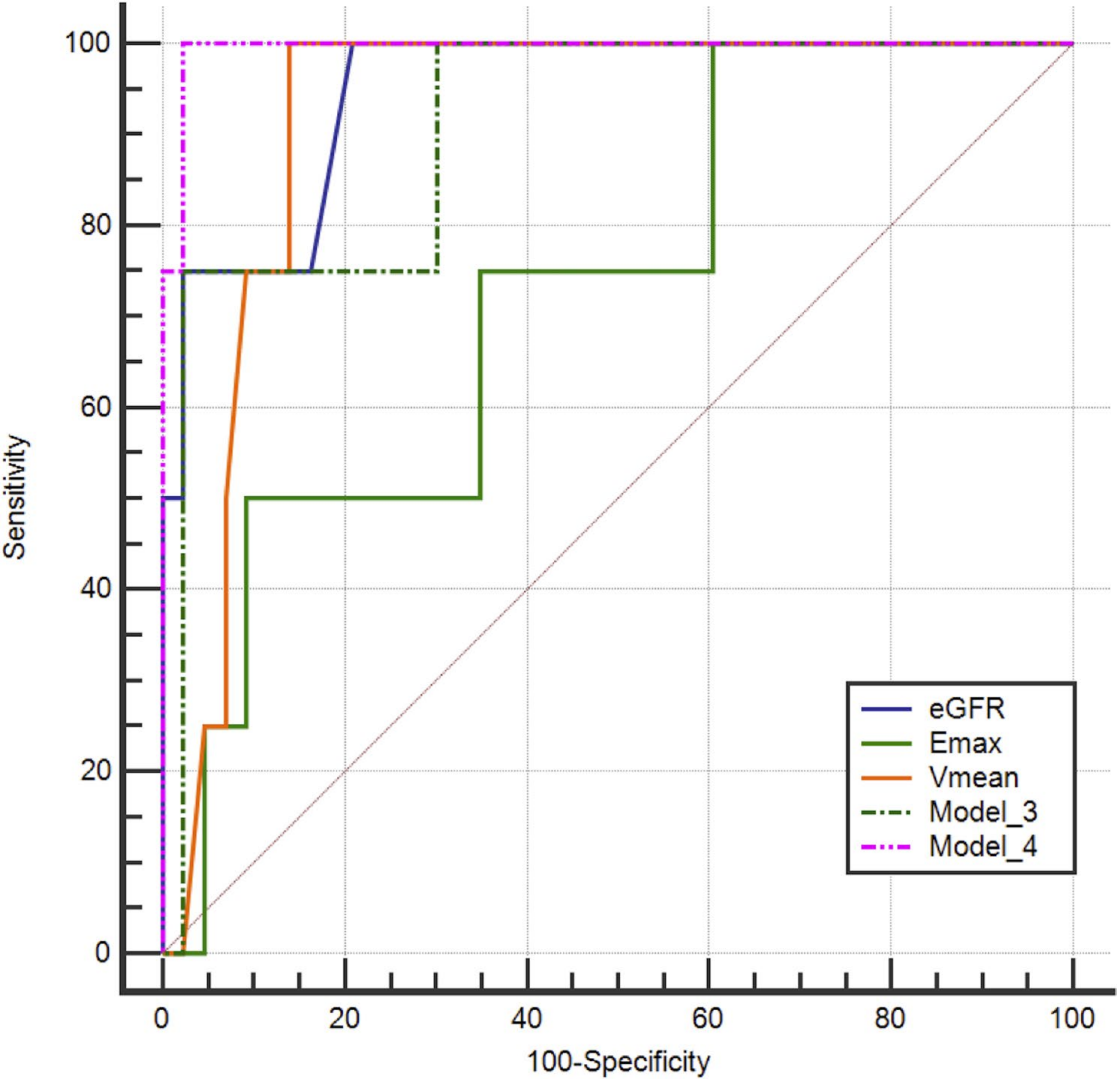
ROC

The AUC of eGFR alone and Vmean alone were excellent and were not significantly different compared with those of Model 4 in predicting severe interstitial inflammation [0.994 (95% CI: 0.913–1.000) vs. 0.948 (95% CI: 0.841–0.991) and 0.994 (95% CI: 0.913–1.000) vs. 0.919 (95% CI: 0.801–0.978), *p* = 0.283 and *p* = 0.060, respectively]. eGFR, Vmean, and Model 4 all had a sensitivity of 100%; however, the specificity of Model 4 was 97.7%, outperforming that of eGFR (*p* = 0.021) and comparable to that of Vmean (*p* = 0.063) (Table 6; Fig. 4).

**Table 6:** Comparison of diagnostic performance in interstitial inflammation between different approaches

Parameters	AUC			Sensitivity			Specificity			Cut-off value
	% (95%CI)	*p* value	*p* value*	(%)	*p* value	*p* value^#^	(%)	*p* value	*p* value^#^	
eGFR	0.948 (0.841–0.991)	0.690	0.283	100	-	-	79.1	0.021*	0.021*	41.00
Emax	0.727 (0.577–0.846)	0.039*	0.054	50.0	-	-	90.7	0.375	0.375	35.50
Vmean	0.919 (0.801–0.978)	0.843	0.060	100	-	-	86.1	0.063	0.063	3.65
Model 3	0.907 (0.786–0.972)	-	0.257	75.0	-	-	97.7	-	1.000	0.38
Model 4	0.994 (0.913–1.000)	-	-	100	-	-	97.7	-	-	0.21

*p* value refers to comparison with Model 3; *p* value^#^ refers to comparison with Model 4. Model 3 included Emax and Vmean, and Model 4 included Vmean and eGFR. AUC, area under the curve; CI, confidence interval; eGFR, estimated glomerular filtration rate; Emax, maximum elasticity; Vmean, mean elasticity. * Represent *p* value < 0.05

Due to the small number of cases in the severe interstitial inflammation group, the sensitivities of each parameter or model were not compared

## Discussion

In this study, we explored the value of viscoelastic mechanical properties in evaluating the severity of fibrosis and interstitial inflammation in CKD. Our results suggested that the combination of eGFR and Emax was an effective tool for distinguishing severe fibrosis from mild-to-moderate fibrosis, with an AUC of 0.902 (95% CI, 0.780–0.969), a sensitivity of 93.3%, and a specificity of 84.4%. Although Vmean did not improve the diagnostic performance of the detection of renal fibrosis grades, it was a potentially useful parameter to detect different grades of interstitial inflammation.

Several studies have confirmed that shear wave elastography plays an important role in assessing liver fibrosis [20, 25–27]. However, the role of elastography in renal fibrosis assessment remains controversial because previous studies have reported inconsistent results. Some studies have shown that renal elastography is positively correlated with the grades of fibrosis [24, 28]. In contrast, other studies have indicated that renal elastography is negatively correlated with the degree of fibrosis [12, 29]. Meanwhile, some studies have suggested that renal elastography is not correlated with fibrosis [13].

The discrepancies in the results of previous studies may be due to several possible reasons. First, anisotropy is normally present in the renal tissue. Therefore, the stiffness value measured when the ultrasound beam was projected parallel to the vasa recta was lower than that when the beam was cast perpendicular to the vasa recta [30]. Second, the renal tissue has an abundant blood vessel distribution. The results of an in vivo study on pigs by Gennisson et al. showed that ligation of the renal artery and vein led to decreased and increased renal elasticity values, respectively [31]. Consistent with this, Warner et al. and Amador et al. also reported similar findings [32, 33], collectively indicating that measured elasticity values are higher in areas with abundant blood flow. However, blood flow in the renal tissue decreases as CKD progresses. Therefore, the increase in stiffness due to fibrosis counteracts the decrease in stiffness due to decreased blood flow. In addition, the kidney is a retroperitoneal organ with a deeper location than that of the liver and a more pronounced beam attenuation. Third, a standard method for measuring renal elasticity is currently lacking. Consequently, previous studies employed different positions, including supine, lateral, and prone, with a varied number of measurements.

Based on the previous literature and our inspection experience, we selected the prone position in this study because it is easier for the operator to acquire valid viscoelastic values in the supine or lateral position, and the kidney is closer to the skin surface than in the anterior position [30]. Additionally, we selected the cortical tissue in the middle of the kidney as the measurement area to minimize the interference caused by anisotropy.

The results of this study revealed that the Emax of the severe fibrosis group was higher than that of the mild-to-moderate fibrosis group. Additionally, the sensitivity of Emax to distinguish mild-to-moderate fibrosis from severe fibrosis was high (100%), however, the specificity was not satisfactory (43.8%). Therefore, a comprehensive model was required to better evaluate renal fibrosis. Accordingly, through multivariate analysis, we found that Emax and eGFR were the most significant variables for predicting severe fibrosis. Consequently, Model 2, which combined Emax and eGFR via the logistic regression algorithm, achieved satisfactory sensitivity and specificity with values of 93.3% and 84.4%, respectively.

Although the AUC of Model 2 was not significantly improved compared with that of eGFR alone, Model 2 had a significantly higher specificity than that of individual eGFR. Meanwhile, Model 2 had a sensitivity comparable to that of the individual eGFR, indicating that the combined use of Emax and eGFR is a more accurate method for predicting the degree of renal fibrosis in most clinical scenarios compared with the individual eGFR.

Subsequently, we also evaluated whether Vmean, a new ultrasonic parameter, could improve the accuracy of fibrosis diagnosis. However, the results suggested that Model 1, consisting of Emax and Vmean, did not show better diagnostic performance than individual Emax. This result is consistent with that of Zhang et al. [20], who showed that DS combined with elasticity did not improve the diagnostic efficacy for liver fibrosis. In contrast, Yuan et al. evaluated the performances of Vmean and Emean in predicting CKD. Their results suggested that Vmean (AUC = 0.900) demonstrated a better predictive efficacy for CKD than Emean (AUC = 0.690) (*p* > 0.05) [22]. The discrepancies between our results and those of Yuan et al. can be attributed to two factors. First, Yuan et al. measured the viscoelastic parameters in the lateral decubitus position, whereas we adopted the prone position in our study. Second, Yuan et al. used Emean as the elastic parameter, whereas our study employed Emax, which may partly explain the differences between our results and those of Yuan et al.

Although the Vmean did not significantly improve the diagnostic accuracy of renal fibrosis in this study, we found that Vmean might be a useful parameter for predicting the inflammatory condition. The individual Vmean had an AUC of 0.919 (95% CI: 0.801–0.978), a sensitivity of 100% and a specificity of 86.1% for predicting the severity of renal interstitial inflammation using. These results are consistent with those of previous studies investigating liver necroinflammation [34, 35]. However, due to the limited sample size of four patients in the severe inflammation group, no reliable conclusions regarding diagnostic accuracy can be drawn.

Additionally, the post-biopsy bleeding rate observed in this study was 27.7%. In contrast, the rates of bleeding complications reported by Monahan et al. and Potretzke et al. were 1.64% and 0.43%, respectively [36, 37]. The higher bleeding rate observed in our study is likely attributable to differences in the definition of hemorrhage. In our protocol, postoperative bleeding was defined as the presence of a perinephric hematoma detected by ultrasound one day after the procedure. In the studies by Monahan et al. and Potretzke et al., bleeding complications were defined as those meeting Common Terminology Criteria for Adverse Events (CTCAE) grade 3 or higher, where grade 3 hemorrhage is defined as bleeding that requires transfusion, an interventional radiology procedure, or an elective operative intervention. According to this definition, only one patient in our study met the criteria for CTCAE grade 3 or higher hemorrhage, resulting in a bleeding complication rate of 2.13%, which aligns closely with the rates reported in their studies. The rate (27.7%) is consistent with those reported in other studies that employed a similar ultrasound-based definition for perinephric hematoma. For instance, Chakrabarti et al. reported perinephric hematoma rates of 46% at 6 h and 36% at 24 h post-biopsy [38]. Similarly, Cildag et al. observed a rate of 38.3% (23/60) on scan performed 24 h after the procedure [39].

Despite the positive outcomes, this study has some limitations that should be considered when interpreting the results. First, the severe inflammation group included only 4 patients (8.5%), representing class imbalance. Therefore, the reported diagnostic accuracy metrics for inflammation detection should be considered preliminary and hypothesis-generating. Second, the single-center design of the study limits generalizability to other populations and settings. Third, all ultrasound measurements were performed by a single operator, preventing assessment of inter-observer reproducibility. Fourth, the absence of detailed grading for fibrosis and interstitial inflammation precluded an analysis of the association between viscoelastic mechanical properties and pathological gradations. Finally, eGFR alone has already demonstrated predictive power for severe fibrosis. Although eGFR combined with Emax achieved a higher specificity, it remains unclear whether the combined use provides better clinical utility. Consequently, future studies should aim for larger sample sizes, multi-center recruitment and multiple operators to confirm these findings and evaluate whether the combined use of eGFR and Emax provides higher clinical utility and net benefit in real-world clinical settings.

## Conclusion

In this study, we observed that although the Vmean did not improve the performance for detecting renal fibrosis, eGFR combined with Emax was a promising tool for distinguishing the degree of renal fibrosis. Nevertheless, the Vmean was a potentially useful parameter for detecting different grades of renal interstitial inflammation. Multicenter studies with larger sample sizes are required to verify this conclusion.

## Data Availability

The datasets used and/or analyzed during the current study are available from the corresponding author on reasonable request.

## Abbreviations

2D-SWE: Two-dimensional shear-wave elastography
AUC: Area under the curve
BMI: Body mass index
CKD: Chronic kidney disease
CI: Confidence interval
CTCAE: Common Terminology Criteria for Adverse Events
DS: Dispersion slope
Emax: Maximum elasticity
Emean: Mean elasticity
Emin: Minimum elasticity
eGFR: Estimated glomerular filtration rate
FPGN: Focal proliferative glomerulonephritis
FSGS: Focal segmental glomerular sclerosis
MCN: Minimal change nephropathy
OR: Odds ratio
PCR: Protein-to-creatinine ratio
RI: Resistive index
ROC: Receiver operating characteristic
ROI: Region of interest
Vmean: Mean viscosity
